# An Unusual Case of Chronic Lymphocytic Leukaemia Involving the Cervix

**DOI:** 10.7759/cureus.21823

**Published:** 2022-02-01

**Authors:** Jack Hamer, Maher Alazizi, Farshad Tahmasebi

**Affiliations:** 1 Obstetrics and Gynaecology, Walsall Manor Hospital, The Walsall Healthcare National Health Service Trust, Walsall, GBR; 2 Obstetrics and Gynaecology, Russells Hall Hospital, The Dudley Group National Health Service Foundation Trust, Dudley, GBR

**Keywords:** small lymphocytic lymphoma (sll), cancer cervix, haematology, gynae oncology, chronic lymphocytic leukaemia

## Abstract

Chronic lymphocytic leukaemia (CLL) is a malignant monoclonal expansion of B lymphocytes, with accumulation of abnormal lymphocytes in the blood, bone marrow, spleen, lymph nodes and liver. It is mainly a disease of the elderly population. Though extra-nodal involvement is common, cervical and vulvovaginal involvement by CLL is particularly uncommon. In this case report, we discuss the case of cervical involvement of CLL in an 84-year-old patient who presented to rapid-access gynaecological clinic following concerns of persistent postmenopausal bleeding. Previously the patient was known to haematology with a well-controlled diagnosis of CLL since 2007. The initial examination was significant for an enlarged, irregular cervix, whereby a punch biopsy was then obtained. Histological analysis revealed evidence of CLL within the cervix.

## Introduction

Chronic lymphocytic leukaemia (CLL) is a neoplastic disease characterised by the malignant monoclonal proliferation and accumulation of small, mature, long-living lymphocytes leading to lymphadenopathy, organomegaly and systemic abnormalities [1]. CLL displays classical immunophenotyping alongside a routine histological infiltration within blood, bone marrow, liver and lymphoid tissue [2].

Though extra-nodal involvement is also common, cervical and vulvovaginal involvement by CLL is particularly uncommon, which can be highlighted by the limited volume of previously published material. In this case report, we discuss the interesting case of cervical involvement of CLL in an 84-year-old patient who presented to rapid-access gynaecological clinic following concerns of persistent postmenopausal bleeding.

## Case presentation

An 84-year-old lady with a past medical history of ischaemic heart disease, chronic kidney disease stage 3, chronic obstructive pulmonary disease and gout presented to the rapid-access gynaecology clinic at a district general hospital. She was referred via the rapid-access pathway by her general physician due to complaints of postmenopausal bleeding, intermittent night sweats and weight loss. The patient had been regularly followed up by her local haematology team with a known diagnosis of CLL, which has been stable for the prior 14 years. As a consequence, the patient had still been on the watch and wait regime from haematology, whereby a prior computerised tomography (CT) scan four months prior to the patient's attendance to gynaecology clinic had confirmed stable CLL.

The patient was assessed within the rapid-access gynaecology clinic. She is Para 3 with all normal vaginal deliveries. The patient is unaware of when she had her last smear test. A bimanual and speculum examination revealed a small retroverted uterus. Additionally, an enlarged and friable cervix was noted with an abnormal appearance. Two lumps were identified at 1 and 4 o’clock position during examination. Subsequently, a punch biopsy was obtained from the largest lump at 4 o’clock position on the cervix.

**Figure 1:**
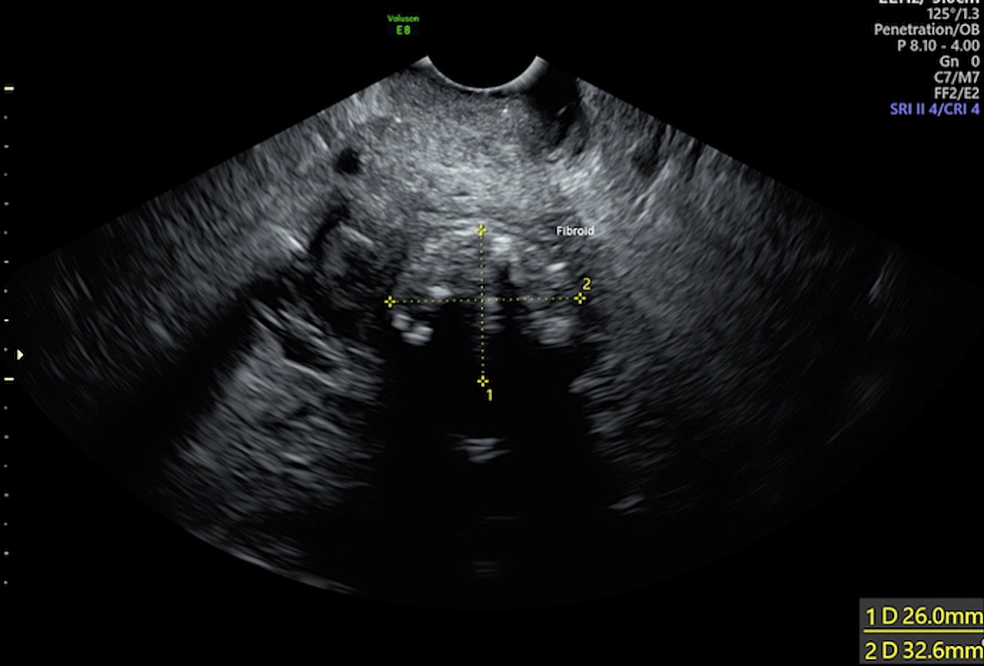
Ultrasound image displaying a small mass in the anterior myometrium measuring 3.2 × 2.6 × 3.4 cm, which represented a calcified fibroid.

**Figure 2:**
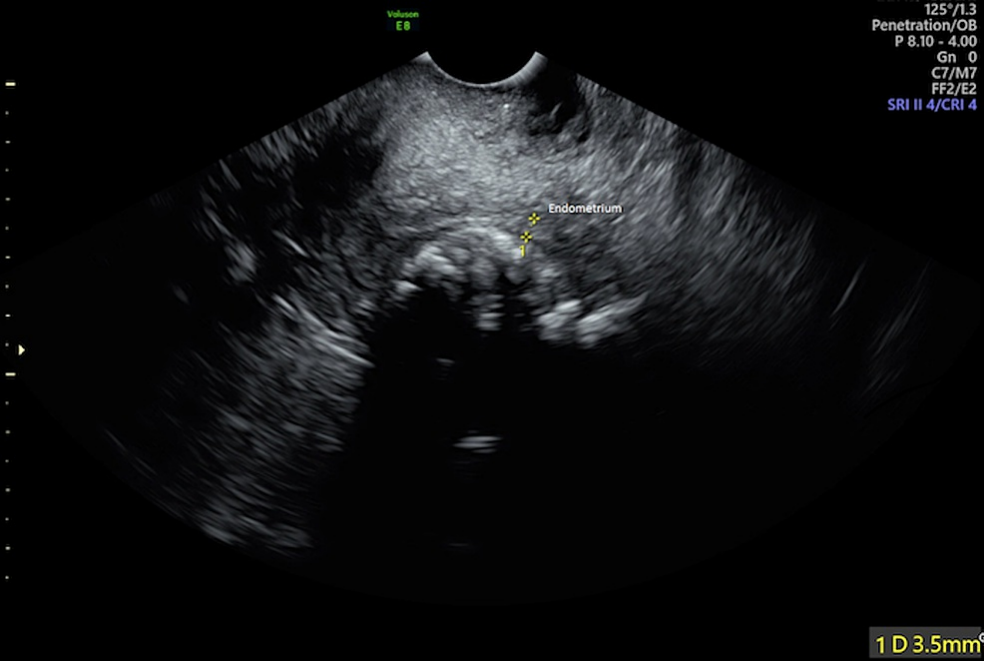
Ultrasound image displaying an endometrial thickness of 3.5 mm with normal adnexa.

A transvaginal ultrasound was performed following attendance in clinic. This displayed a small mass in the anterior myometrium measuring 3.2 × 2.6 × 3.4 cm, which represented a calcified fibroid (Figure 1). Endometrial thickness was 3.5 mm and a normal adnexa was present (Figure 2). 

**Figure 3:**
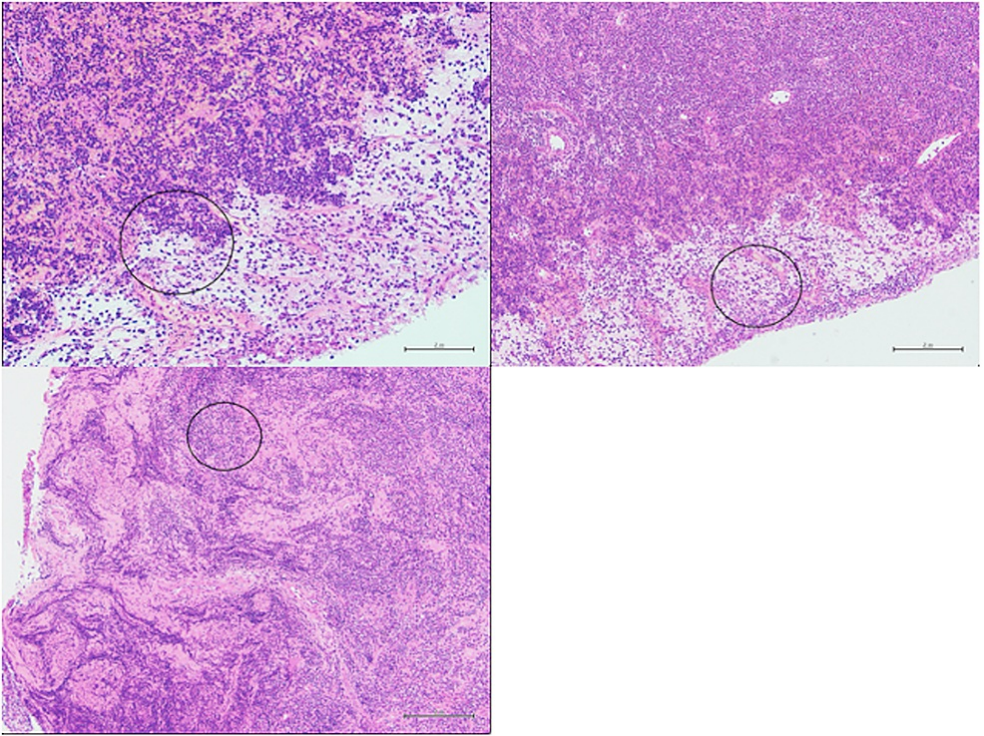
Microscopic findings highlighting diffuse infiltrates of small- to medium-sized lymphoid cells admixed with singly dispersed larger cells with immunoblast-like morphology (present within the circled areas of the images). Additional few foci of ill-defined, indistinct paler-looking areas, possibly represent proliferation centres.

Subsequent results of the biopsy through histological and immunophenotypic analysis is in keeping with small lymphocytic lymphoma (SLL)/CLL as can be seen in Figure 3.

Immunohistochemistry of cells displayed diffuse positive expression with B-cell markers CD20, CD79a and PAX5. There was an additional positive staining with CD21 and CD23, whilst there was a negative staining with CD3, CD10 and cyclin D1. Additionally, co-expression of CD5 and CD43 on B-cells was displayed, whilst LEF1 highlighted patchy focal area with very weak nuclear staining. BCL6 also showed patchy weak staining considered negative. MUM-1 and C-MYC highlighted the paraimmunoblasts. Finally, Ki67 showed a proliferation index of 20-30%.

**Figure 4:**
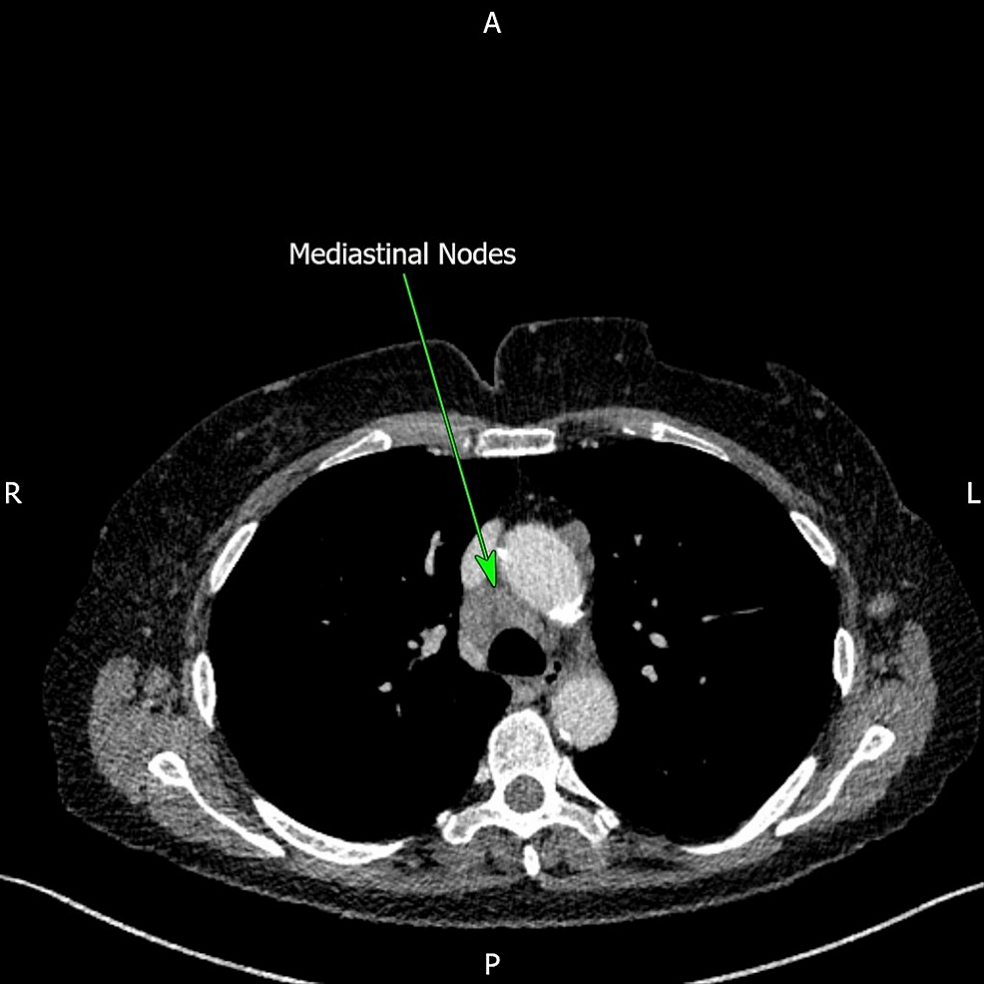
Computed tomography (CT) of the thorax displaying enlarged mediastinal lymph nodes (green arrow).

**Figure 5:**
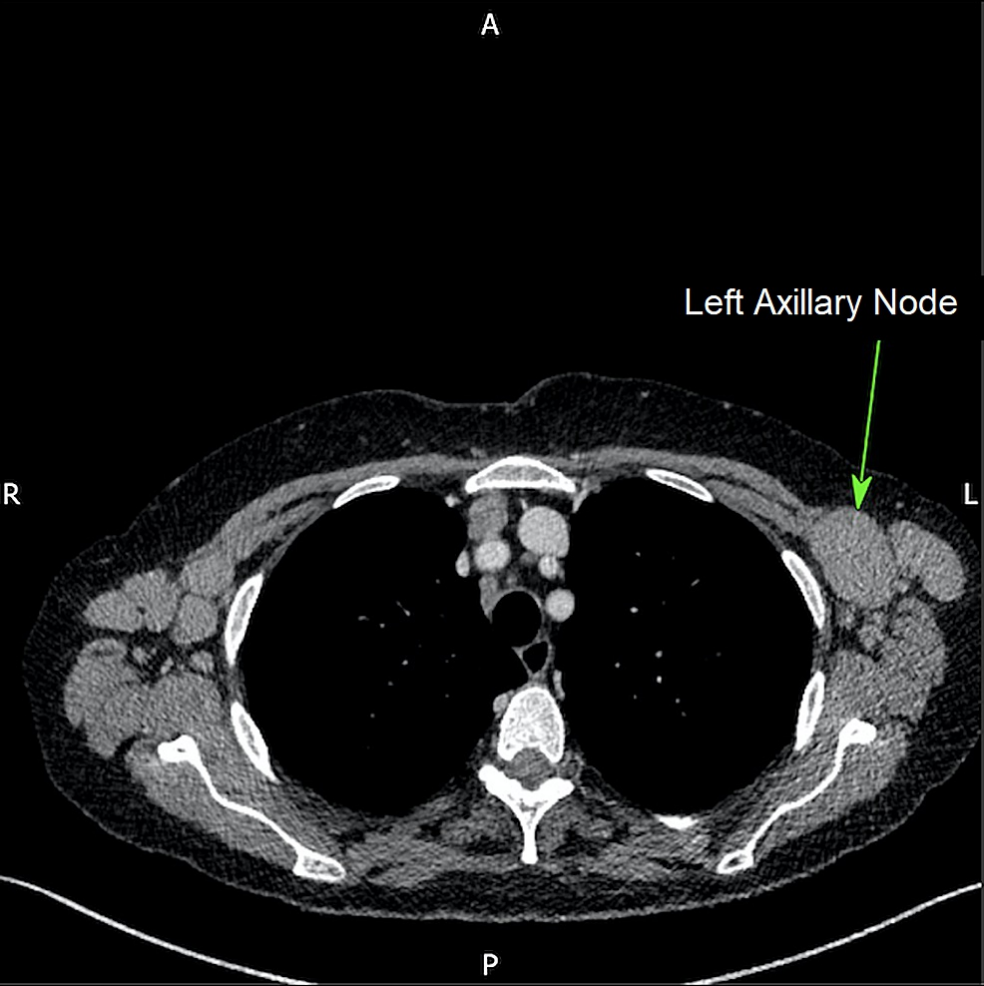
Computed tomography (CT) of the thorax displaying enlarged left axillary lymph nodes (green arrow).

**Figure 6:**
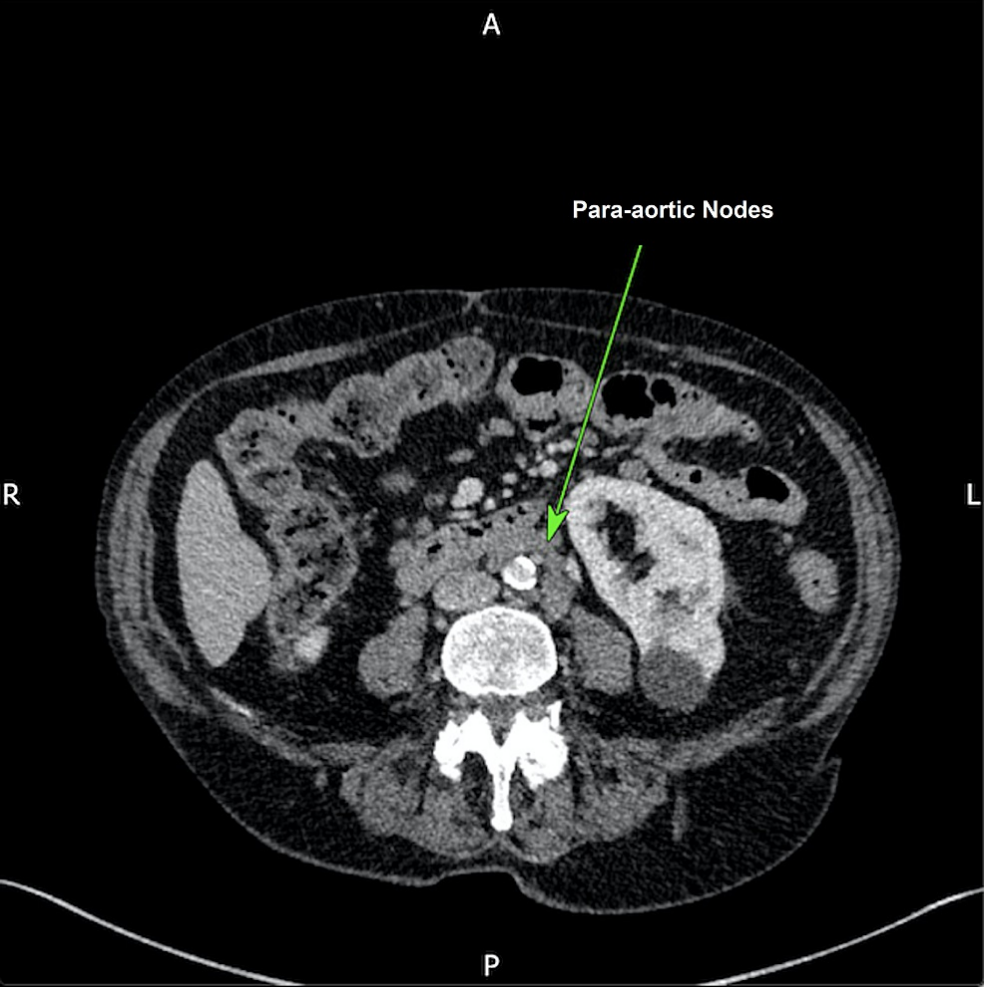
Computed tomography (CT) of the abdomen displaying enlarged para-aortic lymph nodes (green arrow).

**Figure 7:**
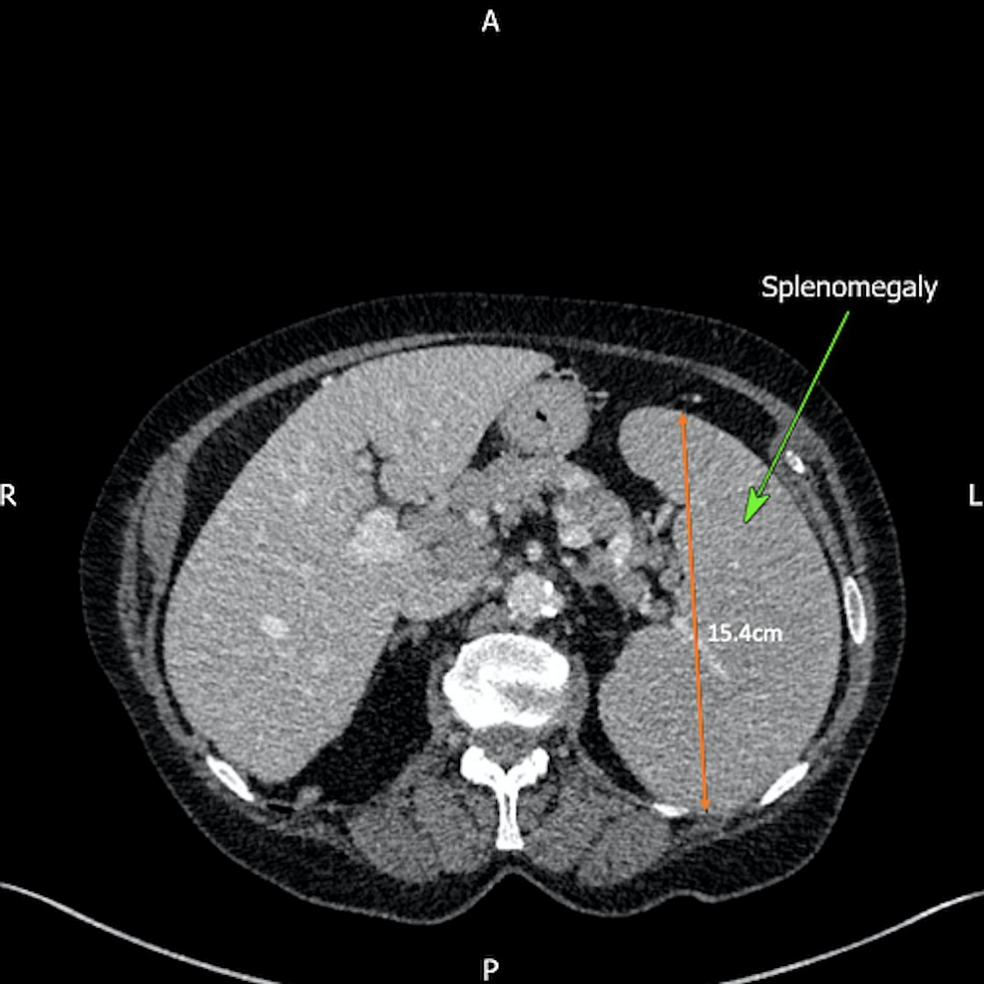
Computed tomography (CT) of the abdomen displaying evidence of splenomegaly (green arrow) with a measurement of 15.4 cm (orange arrow).

Following the findings of SLL/CLL within the cervix, a repeat CT neck, thorax, abdomen and pelvis with IV contrast was arranged by haematology two months after the patient's attendance to the gynaecology clinic. This was following communication between both specialties through a multidisciplinary meeting regarding the patient's care. The CT scan displayed evidence of disease progression with increased lymphadenopathy within the mediastinum, left axilla and para-aortic regions with splenomegaly (15.4 cm) (Figures 4-7).

A month following the CT scan, the patient was seen within the haematology outpatient clinic. Despite the patient's multiple co-morbidities, her performance status was Eastern Cooperative Oncology Group (ECOG) score 1. Haematologists subsequently commenced the tyrosine kinase inhibitor, acalabrutinib, for the patient’s progressive disease. The intention of this treatment was as a curative therapy. The patient responded well to this over the next two months, denying any B symptoms and the per vaginal (PV) bleeding had spontaneously resolved. However, the following month the patient was admitted for a total of eight days for an episode of neutropenic sepsis, although she responded nicely to granulocyte-colony-stimulating factor (GCSF). During this inpatient admission, acalabrutinib was omitted. Whilst the patient felt reasonably well upon discharge, she was noted to have a small degree of oxygen requirement and moderate fatigue. The patient was reviewed within the haematology outpatient clinic two weeks following her inpatient admission. Since the omission of acalabrutinib, the patient noted the return of some, albeit small, lymph nodes in the neck, but denied any B symptoms. Following this clinic review, the patient is now being regularly reviewed within the outpatient haematology clinic, with considerations being made to recommence acalabrutinib. Decisions are to be made as to whether starting acalabrutinib as a lower dose, with intermittent administrations of GCSF, will help minimise her chances of future readmission with neutropenic sepsis. 

## Discussion

Within this particular case, the patient presents with a long history of CLL, which has been stable throughout until detection of a secondary cervical deposit that displays a histological profile associated with CLL/SLL. For close to three decades the World Health Organization have considered CLL and SLL to be near-tissue equivalents, which has been demonstrated within the 2016 revision of the World Health Organization classification of lymphoid neoplasms [3]. Differences may be demonstrated on location of cancer or expression of chemokines [2,4]. Nevertheless, the management of such entities is near the same. This can be through the utilisation of immunochemotherapy agents, either systemic or targeted, as well as possible allogenic haematopoietic stem cell therapy [5-7]. However, new and novel agents are being developed on a more frequent basis.

Gynaecological lymphoblastic infiltration secondary to haematological disorders is rare; however, it is more frequently seen in lymphomas rather than in leukaemias. Thus, we have found the literature available for CLL gynaecological deposits to be scarce [8]. Typically, CLL can have involvement of extra-lymphoid sites within the body, with the skin being the most common site, accounting for approximately 8% of cases [9,10]. Regardless of this, deposits within the female genital tract resulting from primary lymphomas are still particularly rare, resulting in <1% of all extra-nodal lymphomas; however, frequent gynaecological sites tend to be either the ovary or cervix [11,12].

The median age of diagnosis for CLL is 72 years; however, case reports identified through a literature search have displayed a wide age range for patients. Shelke et al. presented a case of a 57-year-old patient with known stage III CLL who presented to the gynaecological department with postmenopausal bleeding [13]. Later analysis revealed a uterine cervix metastasis. In contrast, Seoud et al. demonstrated an evidence of an 82-year-old woman with a known six-year history of stable and non-medicated CLL present with postmenopausal bleeding secondary to CLL deposits with her upper vagina and cervix [14]. Additionally, Magley et al. revealed the case of a 70-year-old lady with a known two-year history of stage IV CLL who was primarily referred to gynaecology following an abnormal cervical smear, which later demonstrated cervical CLL infiltrates [15]. Presented data highlight the evidence that extra-nodal CLL deposits within the cervix can vary with the patients' age, staging of CLL, treatment regime and particularly prognostic factors.

Prior literature has demonstrated the risk of developing a second neoplasm in patients with known CLL to be increased 2.2-fold when associated against a healthy population [16]. In conjunction to this, Schollkopf et al. investigated 12,373 patients with known CLL from 1943 to 2003 for evidence of secondary cancers. Within the cohort, 1,105 secondary cancers occurred, of which 20 involved the female genital tract [17]. Four of the 20 cases originated within the uterine cervix. Despite the rarity of this condition, it is key for the gynaecologists to have a keen awareness of patients presenting with primary gynaecological symptoms with a known haematological malignancy and its possible association to secondary gynaecological metastasis. 

## Conclusions

In summary, this case demonstrates a rare presentation of CLL with secondary cervical involvement causing postmenopausal bleeding. A definitive diagnosis can be made only by histological analysis and immunophenotyping. The case is presented due to its rarity, and to highlight the awareness of the association between haematological and gynaecological malignancies.
